# Propagation of the Madden-Julian oscillation as a deterministic chaotic phenomenon

**DOI:** 10.1126/sciadv.adz1916

**Published:** 2026-02-18

**Authors:** Daisuke Takasuka, Tamaki Suematsu, Hiroaki Miura, Masuo Nakano

**Affiliations:** ^1^ Department of Geophysics, Tohoku University, 6-3 Aramaki-aza-aoba, Aoba-ku, Sendai 980-8578, Japan.; ^2^ RIKEN Center for Computational Science, 7-1-26 Minatojima-minami-machi, Chuo-ku, Kobe 650-0047, Japan.; ^3^ Department of Earth and Planetary Science, The University of Tokyo, 7-3-1 Hongo, Bunkyo-ku, Tokyo 113-0033, Japan.; ^4^ Japan Agency for Marine-Earth Science and Technology, 3173-25 Showa-machi, Kanazawa-ku, Yokohama 236-0001, Japan.; ^5^ Typhoon Science and Technology Research Center, Yokohama National University, 79-5 Tokiwadai, Hodogaya-ku, Yokohama 240-8501, Japan.

## Abstract

The Madden-Julian oscillation (MJO) is a planetary-scale tropical weather disturbance marked by eastward-propagating cumulus cloud clusters over the Indo-Pacific region, causing severe weather and climate events worldwide. The mechanism and predictability of MJO propagation remain elusive, partly because relevant multiscale processes are poorly understood. Here, we reveal chaotic MJO propagation arising from cross-scale nonlinear interactions, based on 4000-member ensemble simulations of two MJO events with a global cloud-system–resolving model. Against conventional linear thinking, multiple regimes with distinct timings of MJO propagation emerge under a single atmosphere-ocean background. The emergence of regime bifurcation depends critically on the equatorial asymmetry of climatological sea surface temperature. Selection of the bifurcated regimes is probabilistic, influenced by whether tropical-extratropical interplay promotes moistening associated with westward-propagating tropical waves over the western Pacific. These results contribute to a more complete MJO conceptual model and help foresee when coherent MJO propagation emerges.

## Teaser

MJO propagation is not described by linear modes but characterized by chaotic behavior with the regime bifurcation.

## INTRODUCTION

The Madden-Julian oscillation (MJO) is the most predominant variability in the tropical atmosphere at intraseasonal timescales, exerting far-reaching impacts on global climate and weather patterns through the substantial subseasonal-scale modulation of equatorial rainfall and large-scale wind fields (*1*). It manifests as an $O\left(10^{3}\right)$-km scale cluster of clouds traveling eastward over the Indo-Pacific region at a speed of around 5 m s^−1^ (*2*). It is called the “storm king” (*3*) and described as “the last type of weather system for which the basic physical mechanisms are not well understood” (*4*). MJO propagation into the tropical western Pacific (WP) has been known to trigger the phase transition of the El Niño/Southern Oscillation (*5*–*7*), awake the Asian/Australian monsoon (*8*, *9*), influence tropical cyclogenesis (*10*), and even affect mid-latitude weather extremes [e.g., heat and cold waves, heavy rainfall, and tornadoes (*11*–*14*)] and stratospheric temperature and circulation in the Arctic (*15*, *16*). With these connections between weather and climate systems via the MJO, understanding MJO propagation is expected to have critical implications for medium-range weather forecasts and climate predictions (*17*).

The eastward propagation of the MJO is often interpreted as a consequence of an east-west asymmetry of moisture fields. This asymmetry is generated intrinsically by MJO-scale dynamics (the equatorial Kelvin-Rossby wave couplet) and climatological mean states (*18*–*21*). In the Indo-Pacific region, where the background moisture gradient is equatorward and eastward, low-level easterlies and cyclonic Rossby gyres associated with MJO convection moisten the free troposphere to its east and dry it to its west (*18*), thereby facilitating the eastward movement of the large cloud system. In addition to this moisture-focused perspective, another emphasizes a dynamical mechanism in which boundary-layer moisture convergence induced by a large-scale Kelvin wave to the east of MJO convection promotes its eastward propagation (*22*, *23*). Either way, these widely accepted views form the basis of representative “linear” MJO theories (*24*–*27*), which consider only MJO-scale perturbations on time-invariant background states and exclude explicit interactions between the MJO and higher-frequency systems. This linearized thinking has been instrumental in evaluating global climate model (GCM) performance (*28*, *29*) and interpreting future climate projections (*30*, *31*). However, it cannot fully explain key observed features, such as the Maritime Continent (MC) barrier effect on MJO propagation (*32*, *33*) and the multiscale structure (*34*, *35*), which are critical for reliable subseasonal-to-seasonal predictions and future projections of MJO-related severe weather.

Here, using huge-ensemble [$O\left(10^{3}\right)$-member] numerical simulations per a single MJO event, we demonstrate that MJO propagation cannot be understood simply by refining theories within the linearized framework; it is more chaotic than previously recognized, originating from cross-scale nonlinear interactions. This notion is underpinned by two notable ingredients: (i) Multiple regimes of MJO propagation emerge under a single background state, depending on climatological sea surface temperature (SST) as an external bifurcation parameter; and (ii) the selection of the bifurcated regime is governed by subtle differences in the cross-scale interactions between high-frequency tropical and extratropical waves and the MJO. Revealing these chaotic features, which are not observable if the MJO is assumed to be a linear system, together with physical processes is made possible by the exascale supercomputer “Fugaku.” Recent advancements in computing power have enabled an increase in high-resolution ensemble members to identify attractors of subseasonal-to-seasonal variability with numerous degrees of freedom, as well as *O*(1–10)-km-scale simulations extending to climate timescales for constructing “Digital Earths” (*36*). The present study relies on the feasibility of the former strategy, leveraging the success of global kilometer-scale modeling for realistic medium-range weather hindcasts (*37*–*39*).

## RESULTS

### Multiple regimes of the MJO propagation

**Fig. 1.**
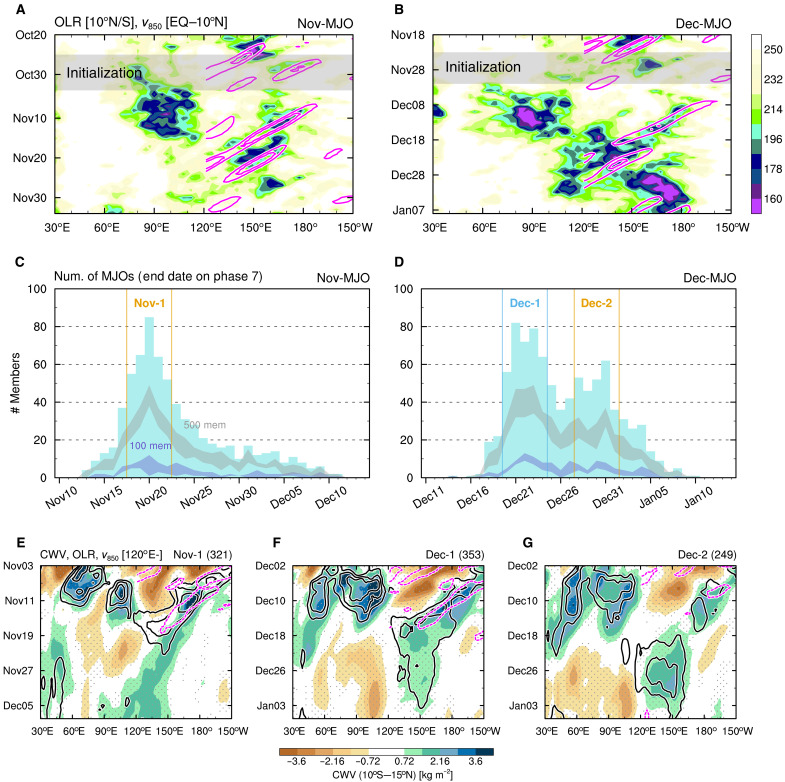
Two MJOs and their regimes revealed by huge-ensemble hindcast simulations. (**A** and **B**) Time-longitude diagrams of observed equatorial (10°S to 10°N) outgoing longwave radiation (OLR) at the top-of-atmosphere (shading) and off-equatorial [the equator (EQ) to 10°N] 850-hPa meridional winds filtered for westward zonal wave numbers 1 to 20 and periods of 5 to 30 days (contours) for Nov-MJO (A) and Dec-MJO (B). Contour interval is 1.6 m s^−1^, with zero values or lower omitted and areas to the west of 120°E masked for visibility. (**C** and **D**) Number distributions by dates of propagation into the WP for Nov-MJO (C) and Dec-MJO (D). Gray (indigo) shading indicates the ranges of the number distributions when using 500 (100) members picked up randomly 100 times. Bins separated by colored vertical lines belong to the three regimes. Num., Number; mem, members. (**E** to **G**) Time-longitude diagrams of equatorial (10°S to 15°N) column water vapor (CWV; shading) and OLR (black) and off-equatorial (EQ to 10°N) meridional wind (magenta) anomalies composited for the Nov-1 (E), Dec-1 (F), and Dec-2 (G) regimes. Black contour interval is 7 W m^−2^, with zero values or higher omitted, and magenta contours represent 0.6 m s^−1^. Stippling indicates statistical significance of shaded values at the 99% level.

With the 14-km mesh Nonhydrostatic Icosahedral Atmospheric Model (NICAM) (*40*, *41*), we conduct $O\left(10^{3}\right)$-member ensemble hindcast simulations for two MJO events that occurred in early November and December 2018 (hereafter referred to as Nov-MJO and Dec-MJO). These events are chosen to assess how two distinct background states—before and after the onset of the Australian monsoon—affect the MJO, under climatologically varying SSTs and land surface temperatures during boreal winter, the MJO’s most active season (*42*). Simulations are initialized with 100 different atmospheric states at 00:00 UTC for each day of a 10-day period, during which MJO convection begins forming over the Indian Ocean (Fig. 1, A and B). These periods are 25 October to 3 November for Nov-MJO and 23 November to 2 December for Dec-MJO, resulting in two subsets of 1000-member simulations each. An additional set of 2000-member simulations is also conducted as sensitivity experiments, in which SST boundary conditions are swapped between the two MJOs to examine a role of SSTs in the regime bifurcation of MJO propagation. All simulations span 45 days, which are sufficiently long to capture the observed eastward migration of the large-scale cloud systems to the WP (around 150°E). This migration occurred around 20 November for Nov-MJO (Fig. 1A) and 23 December for Dec-MJO (Fig. 1B).

This experimental design can generate a wide solution space for the MJO propagation under given boundary conditions. Figure 1 (C and D) shows the frequency distributions of the timing when simulated Nov-MJO and Dec-MJO convection reaches the WP, respectively. For Nov-MJO, the distribution has a single peak at 20 November, when the observed propagation is also completed, albeit with a later-phase tail (Fig. 1A). Namely, the regime that supports Nov-MJO propagation is unique (denoted as Nov-1 regime; Fig. 1C), and this regime is selected with high probability, although its realization remains probabilistic in the model. In contrast, the timing of simulated Dec-MJO propagation bifurcates into two distinct windows: around 22 and 30 December (denoted as Dec-1 and Dec-2 regimes; Fig. 1D). This bimodal distribution is difficult to discern when the ensemble size is limited to 100 or 500, although certain combinations of 500 members can capture it (Fig. 1D). This result reveals that Dec-MJO propagation inherently has the two regimes under a single atmospheric background state prescribed by climatologically varying SSTs—analogous to the bifurcation of solutions in nonlinear dynamical systems (*43*). The observed Dec-MJO propagation can be interpreted as a realization of the Dec-1 regime (in terms of the propagation timing; Fig. 1B).

We create composites of ensemble members for the Nov-1, Dec-1, and Dec-2 regimes to examine their MJO propagation in detail. In the Nov-1 and Dec-1 regimes (Fig. 1, E and F), MJO-scale convective envelopes migrate eastward to 150°E, facilitated by the westward intrusion of moisture that is coupled with off-equatorial 850-hPa meridional wind variations associated with synoptic-scale equatorial Rossby waves from the Pacific (fig. S2). This behavior is consistent with the observed Nov-MJO and Dec-MJO (Fig. 1, A and B), confirming the reliability of the simulations, although the MJO in the Dec-1 regime propagates slightly faster and stagnates longer near 150°E. The possible involvement of westward-propagating waves in MJO propagation was reported in previous studies; some suggested a moistening effect consistent with our finding (*44*, *45*), whereas others highlighted a role as dry intrusion (*33*, *46*). In the Dec-2 regime (Fig. 1G and fig. S2E), the moisture-laden westward-propagating wave decays; however, moisture accumulation over the WP occurs through a different process than in the Dec-1 regime, offering an alternative pathway for MJO propagation.

### Processes supporting the bifurcated regimes of the MJO propagation

**Fig. 2.**
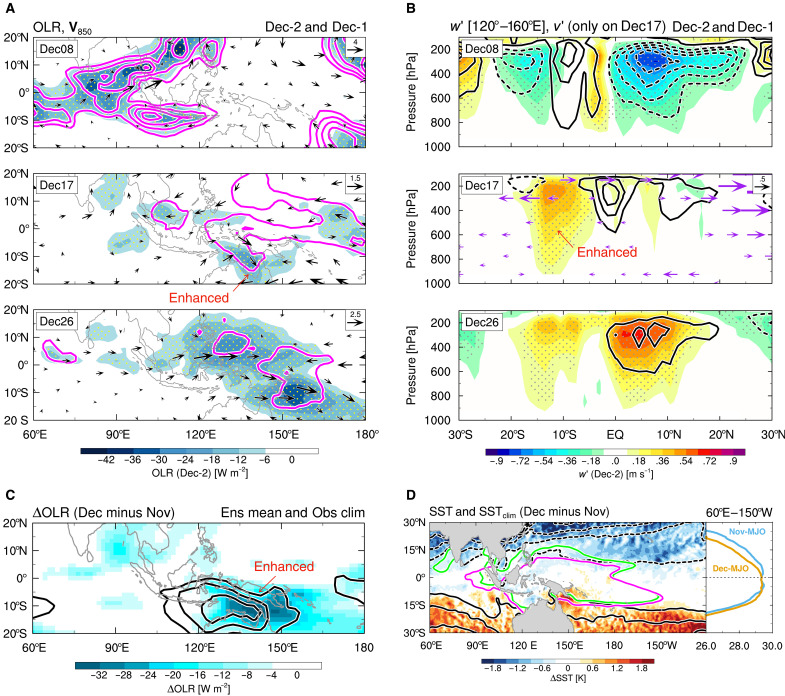
Time evolution of the MJO propagation regimes in December and background effects on it. (**A**) OLR (shading) and 850-hPa wind (vectors) anomalies composited for the Dec-2 regime on 8 December (top), 17 (middle), and 26 (bottom). Contours indicate OLR anomalies for the Dec-1 regime. Contour interval is 12 W m^−2^, with zero values or higher omitted. Stippling indicates statistical significance of color-shaded values at the 99% level. (**B**) As in (A) but for latitude-height cross sections of vertical wind anomalies averaged in 120°E to 160°E. Contour interval is 0.18 m s^−1^, with zero (negative) values omitted (dashed). Meridional wind anomalies for the Dec-2 regime on 17 December are also indicated by vectors. (**C**) Differences (Δ: Dec-MJO minus Nov-MJO) in ensemble and simulation period mean of simulated OLR (shading), and those in simulation period mean of observed climatological OLR (contours). Contour interval is 9 W m^−2^, with zero values or higher omitted. Ens, ensemble; Obs, observed. (**D**) Observed raw ΔSST (shading) and climatological ΔSST (black contours), averaged over the simulation periods. Contour interval is 0.5 K, with zero (negative) values omitted (dashed). Green (magenta) lines indicate the isotherm of 28.5°C in time-mean raw SSTs for Nov-MJO (Dec-MJO). Meridional time-mean SST distributions averaged in 60°E to 150°W are plotted in the right panel.

We first address why the bifurcation of solutions (i.e., multiple regimes) emerges in Dec-MJO propagation. We begin by examining the processes that support the Dec-2 regime, which is the unobserved one. Figure 2A shows the evolution of spatial distributions of outgoing longwave radiation (OLR) and 850-hPa wind anomalies composited for the Dec-2 regime, compared with similar composites for the Dec-1 MJO. While the convective distributions are similar on 8 December, they diverge afterward; on 17 December, Dec-2 MJO convection temporarily weakens over the MC, with its centroid displaced southward (i.e., equatorially asymmetric active convection in the southeastern MC), and then reorganizes over the WP on 26 December, lagging about 1 week behind the Dec-1 regime (see Fig. 1D).

The equatorially asymmetric convective enhancement is unique to the Dec-2 regime and plays a crucial role in its realization. Over the eastern MC, upward motion strengthens in 15°S to 5°S together with lower-level cross-equatorial northerlies on 17 December, preceding the realization of large-scale ascent north of the equator associated with the Dec-2 MJO propagation on 26 December (Fig. 2B). This situation indicates that, even when convection near the equator is inactive, meridional overturning that balances equatorial radiative cooling can be activated if off-equatorial regions favor deep convection. Simultaneously, Ekman balance requires that this meridional circulation induces lower-level easterly (westerly) anomalies in the Northern (Southern) Hemisphere. We identify an anomalous counterclockwise circulation (10°S to 10°N and 120°E to 150°E), directing winds into the equatorially asymmetric active convection in the southeastern MC (Fig. 2A for 17 December). This circulation drives enhanced surface heat fluxes (fig. S3), leading to the subsequent reorganization of convection over the WP. The above evolution contrasts with the Dec-1 regime, where equatorial convection remains active on 17 December because of the moist wave intrusion (see Fig. 1F).

We conjecture that the off-equatorial active convection allowed in the Dec-2 regime is linked to seasonal SST changes from November to December. Compared to the Nov-MJO simulations, the Dec-MJO simulations exhibit enhanced ensemble-mean convective activity in the southeastern MC (Fig. 2C), where the equatorial asymmetry of convection is prominent for the Dec-2 MJO propagation (Fig. 2, A and B), and this region is marked by climatologically active convection during December (Fig. 2C). This corresponds to pronounced SST warming in the Southern Hemisphere, including the region of the active convection, due to the climatological change in December (Fig. 2D). This southward shift of the warm-pool centroid breaks the equatorial symmetry of SSTs, allowing the low-level southward cross-equatorial flow to enhance convection.

### Climatological SSTs as a key to the MJO propagation regime bifurcation

**Fig. 3.**
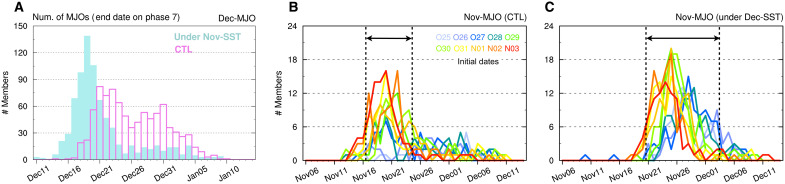
Impacts of seasonal SST components on the timing of MJO propagation. (**A**) Number distributions by dates of propagation into the WP for the Dec-MJO simulation under SSTs used in the Nov-MJO simulation (filled) and for the control Dec-MJO simulation (open). CTL, control. (**B** and **C**) As in (A) but for the control Nov-MJO simulation (B) and the Nov-MJO simulation under SSTs used in the Dec-MJO simulation (C). Results for each 100 members with 10 different initial dates are plotted by different colors.

From the above results, we hypothesize that the seasonal-mean SST controls the presence or absence of the bifurcating regimes in the MJO propagation. Here, we verify this hypothesis using additional huge-ensemble simulations. We first conduct a 1000-member sensitivity experiment for Dec-MJO, in which the oceanic boundary condition is replaced with that used in the Nov-MJO simulation. Under the November-SST condition, the Dec-2 regime does not emerge, and the timing of Dec-MJO propagation is almost uniquely determined, although its realization occurs slightly earlier than the Dec-1 regime (Fig. 3A). As additional support, we also conduct a 1000-member sensitivity experiment for Nov-MJO using SSTs from the Dec-MJO simulation. The timing of Nov-MJO propagation becomes more diverse than in the control experiment with the unique regime (Fig. 3, B and C), and the propagation at a later timing (around 28 November) becomes possible, especially in simulations with long lead times (Fig. 3C). This indicates that the later regime emerges under strong oceanic influences. Together, these results confirm that the bifurcation of the MJO propagation regimes originates from the equatorial asymmetry inherent in the climatological SSTs.

### Tropical-extratropical interaction as a cause of the regime selection

**Fig. 4.**
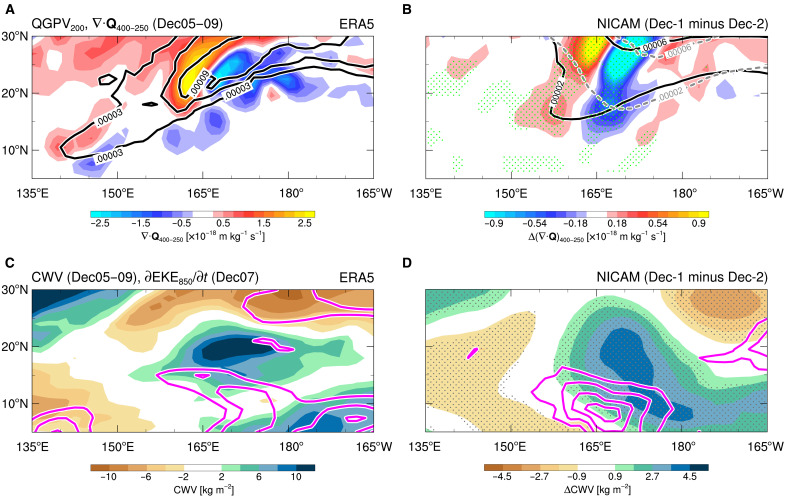
Extratropical influences on the tropical dynamics and thermodynamics related to the regime selection. (**A**) Observed Q-vector divergence in 400- to 250-hPa (shading) and 200-hPa quasi-geostrophic potential vorticity (QGPV; contours) averaged over 5 to 9 December. (**B**) As in (A) but for the simulation. QGPV for the Dec-1 (black solid) and Dec-2 (gray dashed) regimes, and differences ($\Delta_{\text{Dec}}$; Dec-1 minus Dec-2 regime) in the Q-vector divergence are plotted. Stippling indicates statistical significance of shaded values at the 99% level [same for (D)]. (**C**) Observed CWV anomalies averaged over 5 to 9 December (shading), and 850-hPa westward-propagating synoptic-scale EKE tendency on 7 December (contours). Contour interval is 0.28 m^2^ s^−2^ day^−1^, with zero values and lower omitted. (**D**) As in (C) but for $\Delta_{\text{Dec}}$ in the simulations. Contour interval is 0.15 m^2^ s^−2^ day^−1^, with zero values and lower omitted.

As a mechanism explaining the selection of the bifurcated regimes for Dec-MJO propagation, we identify a difference in the strength of the tropical-extratropical interaction between the two regimes. In the observation, an extratropical trough intrudes into the tropical WP in the upper troposphere before the MJO propagation (5 to 9 December; Fig. 4A). This trough forces large-scale ascent to the east, as indicated by mid-to-upper tropospheric Q-vector convergence, which helps moisten the troposphere there (Fig. 4C). These dynamical and thermodynamic processes create conditions conducive to the development and maintenance of the westward-propagating equatorial Rossby wave that leads Dec-MJO propagation (Fig. 1B). This interpretation is supported by the positive tendency of westward-propagating synoptic-scale eddy kinetic energy (EKE) at 850 hPa in 160°E to 180°, along with its budget analysis (Fig. 4C and fig. S4A). Notably, this extratropical wave-breaking–induced MJO propagation has also been reported in other cases (*47*, *48*), not limited to the Dec-MJO event.

The role of the tropical-extratropical interaction in the regime selection is confirmed by the huge-ensemble simulations. The Dec-1 regime, which follows the observed processes of Dec-MJO propagation (Fig. 1F), involves the trough intrusion from the extratropics (black contours in Fig. 4B), consistent with the observations. Meanwhile, this signal is weaker and displaced farther east in the Dec-2 regime (gray contours). Consequently, the Dec-1 regime exhibits a more robust large-scale ascent dynamically forced by the extratropical influences. Consistent with this dynamical difference, the Dec-1 regime also features a moister environment in the off-equatorial region, leading to a stronger positive tendency of synoptic-scale EKE due to enhanced baroclinic conversion (Fig. 4D and fig. S4C). This contrast delineates the watershed between the two regimes: the strong (weak) extratropical influence fosters (inhibits) the development of the westward-propagating equatorial Rossby wave before Dec-MJO propagation, resulting in the Dec-1 (Dec-2) regime.

**Fig. 5.**
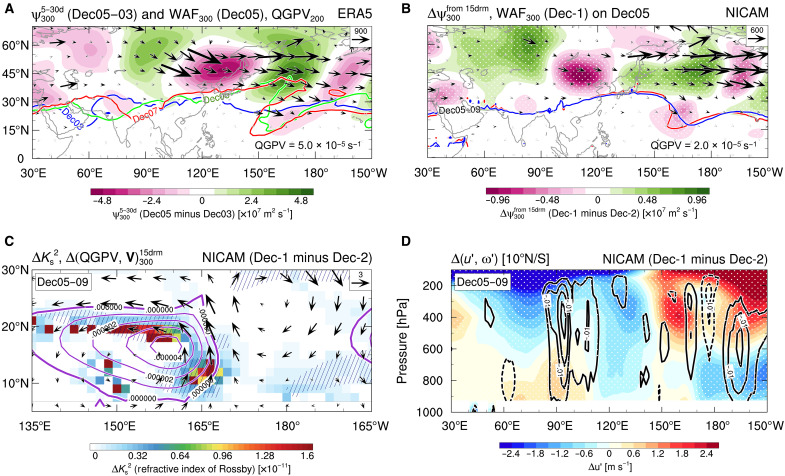
Regime selection by the interaction between the extratropical Rossby wave dynamics and MJO circulation. (**A**) Observed 300-hPa Takaya-Nakamura wave activity flux (WAF) (*69*) on 5 December (vectors); 200-hPa QGPV on 3 December (blue), 5 (green), and 7 (red); and differences (5 December minus 3 December) in 5- to 30-day band-pass-filtered 300-hPa stream function (ψ; shading). d, days. drm, day running mean. (**B**) As in (A) but for the simulation. Shading displays differences in ψ between the Dec-1 and Dec-2 regimes ($\Delta_{\text{Dec}}\psi$) on 5 December. Red (blue) contours indicate time-mean (5 to 9 December; same as below) 200-hPa QGPV for the Dec-1 (Dec-2) regime. Stippling indicates statistical significance of shaded values at the 99% level [same for (D)]. (**C**) Time-mean $\Delta_{\text{Dec}}$ in the refractive index of Rossby waves (shading), 15-day running mean 300-hPa QGPV (per second; contours), horizontal winds (vectors), and QGPV gradients (>2.0 × 10^−12^ s^−1^ m^−1^; hatched). (**D**) Time-mean $\Delta_{\text{Dec}}$ in equatorial (10°S to 10°N) zonal wind (shading) and vertical *p*-velocity (pascals per second; contours) anomalies.

The trough intrusion from the extratropics is a highly transient process, realized as the refraction of extratropical Rossby waves. An observed subtropical jet, measured by quasi-geostrophic potential vorticity (QGPV) at 200 hPa, suddenly meanders in 150°E to 180° on 5 December (green contours in Fig. 5A). Simultaneously, mid-latitude transient Rossby waves disperse energy into the tropics in 150°E to 170°E. This energy dispersion is active in the Dec-1 regime, sustaining stronger cyclonic circulation and more evident trough intrusion in 10°N to 30°N and 150°E to 170°E than the Dec-2 regime (Fig. 5B). In the Dec-1 regime, the higher refractive index of Rossby waves supports clearer refraction of the extratropical Rossby waves, owing to enhanced background QGPV gradients linked to stronger cyclonic circulation in the upper troposphere (Fig. 5C).

The upper-level background cyclonic circulation that influences the extratropical Rossby waves appears to be generated as a Matsuno-Gill response to the MJO convection (*49*), which is active over the western MC at this time. This inspection raises the hypothesis that the intensity of the MJO-related circulations self-regulates the degree of the extratropical-tropical interaction, verified by our simulations. Figure 5D shows the differences in the equatorial zonal circulation before the MJO propagation between the two regimes. As expected, the Dec-1 regime exhibits stronger ascent associated with MJO convection in 90°E to 120°E, accompanied by more predominant upper-level westerlies over the WP. These features constitute the stronger cyclonic circulation shown in Fig. 5C. Summarizing the above results, the differences in the two-way interaction between the extratropical Rossby waves and MJO-scale tropical circulation are responsible for splitting Dec-MJO propagation into the Dec-1 and Dec-2 regimes. Notably, subtle differences in initial MJO amplitudes play a more important role in the regime selection for Dec-MJO propagation than Nov-MJO propagation. This is suggested by the fact that initial amplitudes of the Nov-1 MJO convection are not statistically distinct from those of either Dec-1 or Dec-2 regime (fig. S5).

### Seasonal sensitivity of MJO propagation to the tropical-extratropical interaction

A remaining question is how inevitably the identified tropical-extratropical interaction shapes the MJO propagation regimes in December. Is it possible that the equatorial asymmetry of SSTs not only gives rise to multiple regimes of MJO propagation but also makes MJO propagation more sensitive to the tropical-extratropical interaction? To address this question, we first examine the evolution leading to Nov-MJO propagation (fig. S6). In the observation, extratropical Rossby waves do not refract into the tropics (fig. S6A). In addition, the westward-propagating moisture-laden waves, which contribute to the moistening that supports Nov-MJO propagation (Fig. 1E), are developed by tropical dynamics, more barotropic energy conversion in the lower troposphere than in Dec-MJO for both the observation and simulations (figs. S6, B and C, and S4). These results indicate that the extratropical dynamics does not play a substantial role, at least for the Nov-MJO case. This local eddy-mean flow interaction over the WP is not strongly influenced by convective perturbations in the Indian Ocean, consistent with the weaker sensitivity of Nov-MJO propagation to initial amplitudes (fig. S5).

**Fig. 6.**
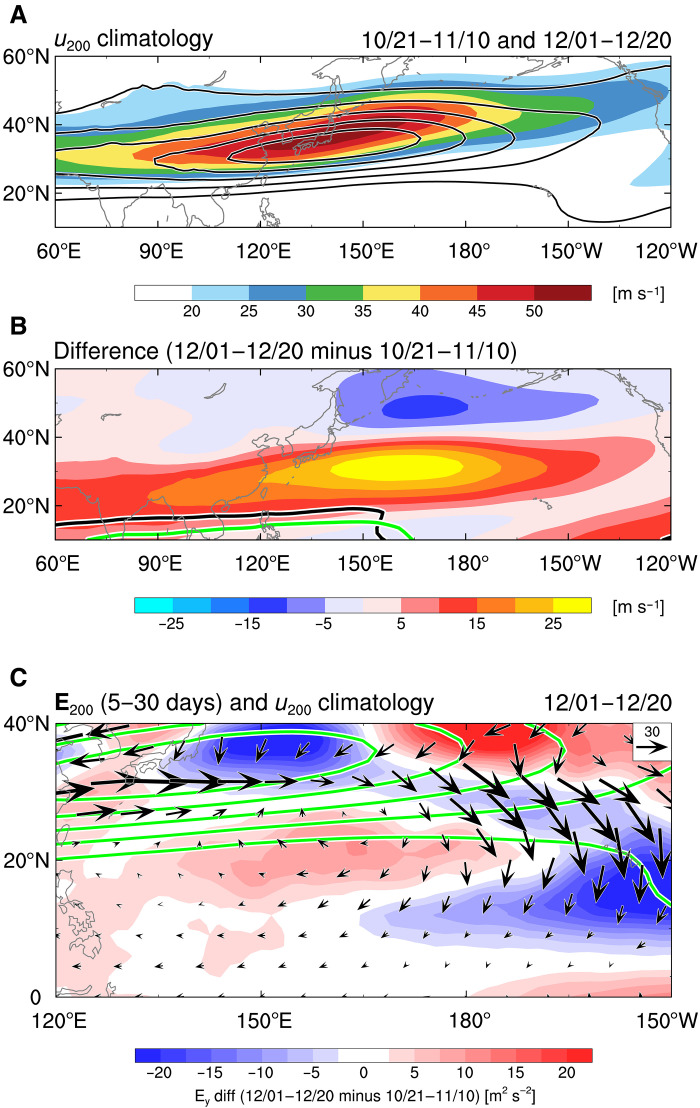
Seasonal differences in the westerly jet and associated eddy propagation. (**A**) Climatological 200-hPa zonal winds during 21 October to 10 November (shading) and 1 to 20 December (contours). Contour interval is 10 m s^−1^, starting from 20 m s^−1^. (**B**) As in (A) but for the differences between the two periods. Black and green contours indicate zero values of 200-hPa zonal winds for 21 October to 10 November and 1 to 20 December, respectively. (**C**) E vectors associated with 5- to 30-day–filtered eddies (vectors) and climatological 200-hPa zonal winds [contours; same as (A)] averaged over 1 to 20 December. Shading represents differences of meridional components of the E vectors between the two periods.

This event-based insight can be generalized by comparing the climatology of the subtropical westerly jet and wave energy propagation between November and December. In December, the westerly jet axis and its exit shift farther equatorward, and upper-level westerlies extend across a broader portion of the tropics (Fig. 6, A and B). This situation implies that December provides extratropical Rossby waves traveling along the westerly jet with more opportunities to influence the tropics. To confirm this view, we examine the climatology of 200-hPa E vectors associated with 5- to 30-day transients in December and their changes from the period including early November (Fig. 6C). The E vectors point toward the tropics near the jet exit and exhibit more equatorward components closer to the equator than in November. This suggests that December has a climatologically higher potential for fostering close coupling between the extratropics and near-equatorial regions. Hence, in December, the degree of the tropical-extratropical interaction is more likely to represent the critical threshold that determines the regime selection of MJO propagation.

## DISCUSSION

If the dynamics of the MJO were essentially linear, as assumed in previous theoretical studies (*24*–*27*), then the bifurcation in the timing of MJO propagation that depends on the seasonal mean SST distributions would never been found. Our huge-ensemble simulations prove that MJO dynamics cannot be explained by a linear theory. Instead, the MJO should be regarded as a nonlinear mode that allows multiple regimes and involves the interactions among the MJO, synoptic-scale equatorial waves, and extratropical disturbances. The physical mechanism we identify as causing chaotic MJO propagation advances the insight from Chen (*50*), who showed chaotic evolution of the MJO amplitude using a Koopman model but left its physical origin an open question.

Our results have implications for the prediction of MJO propagation, which is important for subseasonal-to-seasonal weather forecasts (*17*). The accurate prediction of both Nov-MJO and Dec-MJO propagation depends on skillfully capturing the westward-propagating tropical waves over the WP. The prediction opportunities differ between Nov-MJO and Dec-MJO. For Nov-MJO, they lie in the initial low-level wind fields over the WP, reflecting the importance of local barotropic processes in activating the waves (fig. S6C; contrasting the Nov-1 and nonpropagating members), as well as under the boundary conditions that support the single propagation regime (Figs. 1C and 3A). The SSTs are characterized by moderate El Niño conditions [Oceanic Niño index ≈ 1 K; (*51*)], which enhance MJO realization (*52*) and may thereby increase the probability and predictability of the Nov-1 regime. For Dec-MJO, the opportunities reside in the initial MJO amplitudes; however, the boundary conditions—with equatorial asymmetry superimposed on the moderate El Niño—pose barriers to deterministic prediction of the propagation timing because of the inherent multiple propagation regimes (Fig. 1D). In practice, for this case, sequentially comparing observations and predictions of the strength of MJO convection over the Indian Ocean and the tropical waves in the WP can help improve prediction accuracy. Extending this event-oriented storytelling to other MJOs from different years would provide further insights into the windows of opportunity and barriers for prediction (*53*).

We further provide a perspective for MJO prediction using machine learning (ML). The highly probabilistic nature of MJO propagation revealed here implies limited prediction skill when using ML models trained solely on observational data (*54*) and/or conventional global climate model (GCM) output (*55*, *56*). Observational data alone cannot encompass the full probability space of the MJO, and conventional GCMs struggle to accurately simulate synoptic-scale variations that affect MJO dynamics (*57*). Improved aritificial intelligence–based prediction of the MJO may be achieved by training models on huge-ensemble storm-resolving simulations that capture the bifurcating solutions of MJO propagation arising from the cross-scale interactions.

Our findings also help interpret the seasonality of MJO activity. The enhanced MJO realization from December to February (DJF) (*42*) can be explained by the existence of multiple MJO propagation regimes under equatorially asymmetric SSTs, which become more pronounced during DJF. In line with this view, more than 60% of MJO events in November fail to reach the WP, whereas the failure rate drops to about 25% in DJF (*58*). In our simulations, the MJO fails to propagate in 321 ensemble members for Nov-MJO and in 101 members for Dec-MJO, consistent with the seasonal contrast in propagation likelihood. The higher likelihood of MJO propagation after December is thus possibly associated with a greater variety of propagation regimes with different moistening processes over the WP. Future analyses comparing MJO events across seasons would help clarify how the number of propagation regimes shapes the observed seasonality.

In conclusion, nonlinearity, a necessary condition for chaos, is involved in MJO propagation through moisture advection by synoptic-scale waves. In addition, the bifurcated two regimes, which emerge in response to changes of the equatorial asymmetry of SSTs, transition nonperiodically via the tropical-extratropical interaction. These findings show that MJO propagation can be viewed as a deterministic chaotic phenomenon and that its more skillful prediction necessitates focus on the probabilistic space revealed by huge-ensemble simulations.

## MATERIALS AND METHODS

### Model and simulation setups

In this study, we use the NICAM (*40*, *41*). A set of fully compressible three-dimensional nonhydrostatic dynamical equations is used, and their discretization is on an icosahedral A-grid system with spring dynamics on the sphere (*59*). A globally quasi-uniform horizontal grid interval we adopted is about 14 km. This resolution can represent multiscale structure emerging from cloud systems explicitly and globally, including the MJO (*38*, *60*), although it is still far from a convection-resolving scale. Vertical layers are 38, extending to an altitude of about 40 km above the sea surface. The physics schemes are the same as the configuration used in the “MJO run” in Takasuka *et al.* (*60*).

The atmospheric states in the 1000-member ensemble 45-day simulations for the Nov-MJO and Dec-MJO events are initialized at 00:00 UTC each day during a 10-day period shown in Fig. 1 (A and B). This 10-day period for Nov-MJO and Dec-MJO is 25 October 25 to 3 November and 23 November to 2 December 2018, respectively. The atmospheric initial conditions are obtained from NICAM–Local Ensemble Transform Kalman Filter (LETKF) Japan Aerospace Exploration Agency (JAXA) Research Analysis (NEXRA) dataset (*61*), which has 100 ensemble members every 6 hour with a horizontal resolution of 1.25° × 1.25°. We use all the 100 members of NEXRA at 00:00 UTC during the above periods (10 days × 2), and thus, we can create the two sets of the 1000-member simulation as the control hindcast experiments for Nov-MJO and Dec-MJO. The oceanic state is initialized by the National Oceanic and Atmospheric Administration (NOAA) Optimum Interpolation Sea Surface Temperature (OISST) Version 2.1 (*62*), and the oceanic boundary conditions of SST and sea ice fraction are given by the linear temporal interpolation of the weekly OISST data. The land initial condition is the monthly mean climatology of the NICAM simulation with a 220-km horizontal mesh. These oceanic and land data are the same for all the ensemble simulations.

In addition to the control hindcast experiments, we conduct the two sets of the 1000-member ensemble sensitivity experiments to examine the impacts of SST distributions on the behavior of solutions in the MJO propagation. In one experiment, the oceanic initial and boundary conditions used in the Dec-MJO control simulations are replaced with those from the Nov-MJO control simulation. Specifically, this 1000-member simulation spans the calendar period from 23 November 2018 to 16 January 2019, while the oceanic conditions are taken from 25 October to 18 December 2018. In the other experiment, this relationship between the calendar period and oceanic conditions is reversed. All the configurations other than the oceanic conditions are identical to those in the control simulations.

### Observational data

We use the three observational datasets. First, interpolated daily OLR obtained from the NOAA polar-orbiting satellite (*63*) is used as a proxy for deep convective activities. The horizontal resolution is 2.5° × 2.5°. The data cover the period from January 1979 through December 2019 and used for constructing phase space that describes the time evolution of MJO convection (described later) and for checking the observed Nov-MJO and Dec-MJO propagation. We also use 6-hourly snapshots from the European Centre of Medium-range Weather Forecasts (ECMWF) fifth-generation global reanalysis (ERA5) data (*64*) with a horizontal resolution of 1.5° × 1.5°, averaged to daily values that span the period from January 1979 to December 2022. We depict the three-dimensional variables of zonal and meridional winds (*u* and *v*), vertical *p*-velocity (ω), temperature (*T*), specific humidity ($q_{v}$), and geopotential (Φ). The three-dimensional data used for the analyses have 15 pressure levels, spanning from 1000 to 50 hPa. In addition, we take the two-dimensional surface pressure data to calculate column-integrated water vapor content from the surface to 100 hPa. Daily anomalies are calculated by the removal of the first three harmonics of daily climatologies for 1979 to 2022, before we apply spatial and/or temporal filtering to any variables. Last, we use the observed daily SST data from OISST Version 2.1 (*62*). The daily climatology used in Fig. 2D is calculated from the 1971–2000 base period. The horizontal resolution is 0.25° × 0.25°.

### Identifying the MJO propagation and its composite

To identify the completion of the MJO propagation in our ensemble simulations, we monitor the time evolution of the MJO index introduced by Kikuchi *et al.* (*65*). The MJO index is calculated for every ensemble member. Originally, the MJO index is constructed by two leading principal components (PC1 and PC2) obtained from the extended empirical orthogonal function (EEOF) analysis for the long-term daily OLR data in the tropics. The period of our simulations, however, is only 45 days, which are not sufficiently long to calculate the statistically robust EEOFs. Thus, we adopt the hybrid usage of the simulation and observational data to obtain the MJO index here. This treatment has been taken similarly in a previous study (*66*). The specific procedure is as follows:

1) We calculate two leading EEOFs (EEOF1 and EEOF2) of observed intraseasonal OLR anomalies in the tropics (30°S to 30°N and 0° to 360°E) from DJF in 1979 to 2019. Here, intraseasonal OLR anomalies are obtained by applying Lanczos band-pass filter (*67*) with 25- to 90-day cutoff periods and 201 weights to daily unfiltered OLR anomalies. The daily unfiltered OLR anomalies are calculated by the removal of the first three harmonics of daily climatology for 1979 to 2019. The EEOF analysis uses three time-lagged data: days −10, −5, and 0.

2) To calculate the MJO index for every ensemble member, we prepare for the simulation-based daily OLR data projected onto the two observation-based EEOFs (denoted as EEOFs^Obs^). We first subtract the time mean of the simulated OLR data during the entire period of the simulation for each member (denoted as OLRA^Sim^). If OLRA^Sim^ are projected onto EEOFs^Obs^ with the time lags (days −10, −5, and 0), then we cannot obtain the MJO index on the first 10 simulation days. Thus, the 10-day time series of observed OLR anomalies (denoted as OLRA^Obs^) are appended before the beginning of the time series of OLRA^Sim^. For instance, for a 45-day time series of OLRA^Sim^ that starts at $t=t_{0}$ [day], OLRA^Obs^ from $t=t_{0}-10$ to $t=t_{0}-1$ are attached. Here, OLRA^Obs^ are defined as the deviations from the time mean during the simulation period (i.e., $t_{0}\leq t\leq t_{0}+45$) for the consistency of the base period of the anomalies with OLRA^Sim^.

3) We project the 55-day OLR data created above onto the two EEOFs^Obs^, yielding the 45-day time series of PC1 and PC2. Then, we apply 5-day running mean to these two PCs to remove the high-frequency noisiness and define the amplitudes ($A=\sqrt{\text{PC}1^{2}+\text{PC}2^{2}}$) and phases [$\alpha ={\text{tan}}^{-1}\left(\text{PC}1/\text{PC}2\right)$] of the MJO index.

Next, we track the day-to-day evolution of the amplitudes and phases of the MJO index to identify when the MJO convection completes its propagation into the WP. The phases are divided into eight (phases 1 to 8) by π/4 phase angle (see fig. S1; the consecutive phase progression captures the eastward propagation of large-scale convective envelopes successfully). We impose four criteria on the tracking of the MJO index:

1) Passing through phase 1 within the first 20 days.

2) *A* > 0.4 during tracking, except that the number of days with *A* ≤ 0.4 is 4 days or less.

3) No more than one phase skipping, and no more than three phase recession.

4) Completion of tracking up to phase 7 or 8 (i.e., from phase 6 to 7, 5 to 7, and 6 to 8), or that of staying at phase 6 for more than 7 days with all the above conditions satisfied.

The first criterion ensures the robust MJO initiation over the Indian Ocean, the second and third ones require the eastward migration of the MJO structure with certain strength, and the fourth criterion confirms the MJO propagation into the WP. The timing of the MJO propagation into the WP (see Fig. 1, C and D) is specified as the day on which the amplitudes take the maximum, of those that meet the fourth criterion.

After identifying the MJO propagation timing, we apply the composite analyses to the ensemble members categorized into the three representative regimes of the MJO propagation: the Nov-1, Dec-1, and Dec-2 regimes. Unless otherwise noted, the anomalies of any variables used to create composites are defined as deviations from the simulation period mean for each ensemble member. Statistical significance of the composite anomalies and their differences between the regimes is assessed by a two-tailed Student’s *t* test, on the assumption that the different time evolutions among the ensemble members are statistically independent.

### Moist static energy budget

We conduct the column-integrated moist static energy (MSE) budget analysis to understand the moistening processes responsible for the MJO propagation into the WP. This relies on the fact that the MSE tendency explains tropical moisture variations well because the horizontal temperature gradient is weak in the tropics. The budget equation used here is

$$\langle \partial_{t}h\rangle =-\langle v_{h}\cdot \nabla h\rangle -\langle \omega \partial_{p}h\rangle +\text{SHF}+\langle Q_{R}\rangle$$ (1)

where $h=C_{p}T+\Phi +L_{v}q_{v}$ is MSE ($C_{p}=1004.69$ J kg^−1^ is the specific heat at constant pressure; and $L_{v}=2.50084$ × 10^6^ J kg^−1^ is the latent heat for vaporization), $v_{h}$ is the horizontal velocity vector, SHF is the sum of surface latent and sensible heat fluxes, and $\langle Q_{R}\rangle$ is radiative heating. The angle brackets indicate mass-weighted vertical integration from the surface to 100 hPa. $\langle Q_{R}\rangle$ is calculated as the difference of radiative fluxes between the top of the atmosphere and the surface. The budget terms are evaluated with 6-hourly values, and then they are averaged to the daily values.

### Eddy kinetic energy budget

The EKE budget is analyzed to explain processes that affect the equatorial Rossby wave activities involved in the MJO propagation. The budget equation is

$$\begin{array}{c} \frac{\partial \bar{K^{\prime }}}{\partial t}=-\underset{\text{KmKe}}{\underbrace{\bar{v_{h}^{\prime }\left(v^{\prime }\cdot \nabla \right)v_{h}}}}-\underset{\text{AmKe}}{\underbrace{\bar{v}\cdot \nabla \bar{K^{\prime }}}}\\ -\underset{\text{AeKe}}{\underbrace{\bar{v^{\prime }}\cdot \nabla \bar{K^{\prime }}}}-\underset{\text{PeKe}}{\underbrace{\frac{R}{p}\bar{\omega^{\prime }T^{\prime }}}}-\underset{\mathrm{GKe}}{\underbrace{\bar{\nabla \cdot \left(v^{\prime }\Phi^{\prime }\right)}}}+\left(\text{Residual}\right) \end{array}$$ (2)

where $K^{\prime }=\left({u^{\prime }}^{2}+{v^{\prime }}^{2}\right)/2$ is EKE, v is the three-dimensional wind vector, and R=287.05 J kg^−1^ is the dry gas constant. The terms on the right-hand side are physically explained as follows: *KmKe* is the barotropic conversion from mean flows to EKE; *AmKe* and *AeKe* are the EKE advection by mean and eddy flows, respectively; *PeKe* is the baroclinic conversion from the eddy available potential energy; *GKe* is the EKE dispersion via the work done by pressure gradient forces; and Residual includes diffusive processes. *KmKe*, *PeKe*, and Residual correspond to source and sink terms, and the others contribute to the redistribution of EKE.

For the observations (simulations), primes and overbars denote values filtered for zonal wave numbers −20 to −1 and periods of 5 to 30 days (deviations from 11-day running mean) and 11-day running mean, respectively. The definition of the primes differs between the observations and simulations because the simulation data period is too short for filtering. However, this is not an issue, as both definitions successfully capture variations associated with the equatorial Rossby waves (Fig. 1, B and E to F). To prevent the loss of the simulated data at their edges when calculating the running mean, we append the observational data before and after the simulation periods before the budget calculation. For filtering, we use fast Fourier transforms in space and a 201-point Lanczos band-pass filter (*67*) in time.

### Diagnoses based on QG dynamics

We provide some diagnoses based on QG dynamics to understand the tropical-extratropical interaction as a cause of the regime selection of the Dec-MJO propagation. Here, we describe the methodologies used to calculate the four quantities: QGPV, Q-vector, the phase-independent wave activity flux, and the refractive index of Rossby waves. In this study, we adopt the β-plane approximation with a reference latitude 35°N for all the QG diagnoses. The wave activity flux and refractive index of Rossby waves are calculated on the basis of the composite fields, not on individual ensemble members. This can capture the behavior of Rossby waves directly related to the overall features of the regimes of interest.

### Definition of the QGPV

The QGPV on the pressure coordinate is defined as

$$q=f_{0}+\beta y+\frac{\partial^{2}\psi }{\partial x^{2}}+\frac{\partial^{2}\psi }{\partial y^{2}}+\frac{\partial }{\partial p}\left(\frac{f_{0}^{2}}{S^{2}}\frac{\partial \psi }{\partial p}\right)$$ (3)

where $f_{0}$ is the Coriolis parameter at the reference latitude (35°N), $S^{2}=-\alpha \left(\partial \text{ln}\theta /\partial p\right)$ is the static stability parameter (α is the specific volume and θ is the potential temperature), and $\psi =\left(\Phi -\Phi_{\text{ref}}\right)/f_{0}$ is the QG stream function. We use $S^{2}$ and $\Phi_{\text{ref}}$ calculated as their time mean (1979 to 2022 and the simulation period for the observations and simulations, respectively) and areal mean over the Northern Hemisphere, so they are the functions of only *p*. To remove small-scale variations that do not follow the geostrophic motion largely, we apply nine-point spatial smoothing to the obtained QGPV.

### Q-vector analysis

We calculate the divergence of the **Q**-vector (*68*) to diagnose the vertical motions forced by the QG dynamics. Horizontal components of the **Q**-vector are represented by

$$Q\equiv -\frac{R}{p}\nabla_{h}v_{\text{gh}}\cdot \nabla_{h}T=f_{0}\left(\begin{array}{c} -\psi_{\mathrm{xy}}\psi_{\mathrm{xp}}+\psi_{\mathrm{xx}}\psi_{\mathrm{yp}}\\ -\psi_{\mathrm{yy}}\psi_{\mathrm{xp}}+\psi_{\mathrm{xy}}\psi_{\mathrm{yp}} \end{array}\right)$$ (4)

where $\nabla_{h}$ is the horizontal gradient operator and $v_{\text{gh}}$ is the horizontal velocity vector associated with geostrophic motions.

### Calculation of the phase-independent wave activity flux

We use the phase-independent wave activity flux introduced by Takaya and Nakamura (*69*) to evaluate the Rossby wave energy dispersion. We consider the contributions from both stationary and migratory QG eddies on a pressure level. The equation of the corresponding wave activity flux is as follows

$$W_{h}=\frac{1}{2|\bar{v_{h}}|}\left[\begin{array}{c} \bar{u}\left({\psi_{x}^{\prime }}^{2}-{\psi^{\prime }\psi^{\prime }}_{xx}\right)+\bar{v}\left(\psi_{x}^{\prime }\psi_{y}^{\prime }-{\psi^{\prime }\psi^{\prime }}_{xy}\right)\\ \bar{u}\left(\psi_{x}^{\prime }\psi_{y}^{\prime }-{\psi^{\prime }\psi^{\prime }}_{xy}\right)+\bar{v}\left({\psi_{y}^{\prime }}^{2}-{\psi^{\prime }\psi^{\prime }}_{yy}\right) \end{array}\right]+C_{\bar{v_{h}}}M$$ (5)

Here, $C_{\bar{v_{h}}}\equiv C_{P}{\left(u/|v_{h}|,v/|v_{h}|\right)}^{T}$ is the horizontal vector that represents the wave phase propagation projected onto the direction of $\bar{v_{h}}$ (the horizontal vector of background winds) and M=1/2(A+E) with $A={q^{\prime }}^{2}/\left(2|\nabla_{h}\bar{q}|\right)$ and $E=e/\left(|\bar{v_{h}}|-C_{P}\right)$, where $C_{P}$ is the wave phase speed in the direction of $|\bar{v_{h}}|$; and *e* is the wave energy, represented as

$$e=\frac{1}{2}\left[{\left(\frac{\partial \psi^{\prime }}{\partial x}\right)}^{2}+{\left(\frac{\partial \psi^{\prime }}{\partial y}\right)}^{2}+\frac{f_{0}^{2}}{S^{2}}{\left(\frac{\partial \psi^{\prime }}{\partial p}\right)}^{2}\right]$$ (6)

We apply four times of nine-point spatial smoothing to *q* and $\bar{q}$ when calculating *A*. As in the EKE budget analysis, the background states (overbars) and perturbations (primes) are defined differently between the observations and simulations because of the limitation of the simulated data period. For the observations, the overbars and primes denote values filtered for periods of 30 days and more and 5 to 30 days, respectively. Meanwhile, the overbars and primes for the simulated data indicate 15-day running mean and deviations from it, respectively.

To calculate the wave activity flux from migratory QG eddies (i.e., $C_{\bar{v_{h}}}M$), we estimate $C_{P}$ at each grid point, following Takaya and Nakamura (*69*). First, on the day of interest ($t_{0}$), we first compute the two correlation coefficients of $\Phi^{\prime }\left(t\right)$ $\left(t_{0}-10\leq t\;\left[\text{day}\right]\leq t_{0}+10\right)$ between a base point and other grids within a 40° × 40° grid box centered at the base point, imposing a lag of −1 day and 1 day. Then, using the created one-point correlation maps, we trace the grid point with the maximum positive correlation from the negative to positive lag to estimate the actual wave phase propagation. Last, the horizontal vector of the actual phase propagation at the local point is projected onto the direction of local background winds ($|\bar{v_{h}}|$), and then $C_{P}$ is estimated locally as the magnitude of the projected vector.

### Refractive index of Rossby waves

Following Nishii and Nakamura (*70*), we calculate the refractive index of Rossby waves as follows

$$K_{s}^{2}=\frac{|\nabla_{h}\bar{q}|}{|\bar{v_{h}}|-C_{P}}-\frac{f_{0}^{2}}{4N^{2}H_{0}^{2}}\left(1-4H_{0}N\frac{{\mathrm{dN}}^{-1}}{{\mathrm{dz}}^{*}}+4H_{0}^{2}N\frac{d^{2}N^{-1}}{{\mathrm{dz}}^{*2}}\right)$$ (7)

where *N* is the Brunt-Vaisala frequency averaged over the Northern Hemisphere and simulation period, $H_{0}$ is the scale height (set to 8.5 km), and $z^{*}=-H_{0}\text{ln}\left(p/p_{0}\right)$ (i.e., log-pressure vertical coordinate). The overbars indicate 15-day running mean. As in the calculation of the wave activity flux, four times of nine-point spatial smoothing are applied to the $\bar{q}$ field.

### E vector analysis

We compute the **E** vector (*71*) from the ERA5 daily fields to examine observational differences in the climatological characteristics of the eddy shape and propagation between November and December. The **E** vector is defined as

$$E=\left(\bar{{v^{\prime }}^{2}-{u^{\prime }}^{2}},\bar{-u^{\prime }v^{\prime }}\right)$$ (8)

where the overbars and primes denote the daily climatology in 1979 to 2022 and values filtered for periods of 5 to 30 days, respectively. Here, we compare two periods: 21 October to 10 November and 1 to 20 December, corresponding to the periods before and after the seasonal transition between November and December.

## References

[1] C. Zhang, Madden-Julian oscillation: Bridging weather and climate. Bull. Am. Meterol. Soc. 94, 1849–1870 (2013).

[2] R. A. Madden, P. R. Julian, Description of global-scale circulation cells in the tropics with a 40–50 day period. J. Atmos. Sci. 29, 1109–1123 (1972).

[3] E. Hand, The storm king. Science 350, 22–25 (2015). 26430097 10.1126/science.350.6256.22

[4] D. A. Randall, *Atmosphere, Clouds, and Climate* (Princeton Univ. Press, ed. 1, 2012).

[5] M. J. McPhaden, Genesis and evolution of the 1997–98 El Niño. Science 283, 950–954 (1999). 9974381 10.1126/science.283.5404.950

[6] Y. N. Takayabu, T. Iguchi, M. Kachi, A. Shibata, H. Kanzawa, Abrupt termination of the 1997–98 El Niño in response to a Madden–Julian oscillation. Nature 402, 279–282 (1999).

[7] H. H. Hendon, M. C. Wheeler, C. Zhang, Seasonal dependence of the MJO–ENSO relationship. J. Clim. 20, 531–543 (2007).

[8] T. Yasunari, Cloudiness fluctuations associated with the Northern Hemisphere summer monsoon. J. Meteor. Soc. Jpn. 57, 227–242 (1979).

[9] H. H. Hendon, B. Liebmann, A composite study of onset of the australian summer monsoon. J. Atmos. Sci. 47, 2227–2240 (1990).

[10] E. D. Maloney, D. L. Hartmann, Modulation of eastern North Pacific hurricanes by the Madden–Julian oscillation. J. Clim. 13, 1451–1460 (2000).

[11] S. B. Cerne, C. S. Vera, Influence of the intraseasonal variability on heat waves in subtropical South America. Clim. Dyn. 36, 2265–2277 (2011).

[12] J.-H. Jeong, C.-H. Ho, B.-M. Kim, W.-T. Kwon, Influence of the Madden-Julian oscillation on wintertime surface air temperature and cold surges in East Asia. J. Geophys. Res. Atmos. 110, 10.1029/2004JD005408 (2005).

[13] C. Jones, L. M. Carvalho, Spatial–intensity variations in extreme precipitation in the contiguous United States and the Madden–Julian oscillation. J. Clim. 25, 4898–4913 (2012).

[14] D. B. Thompson, P. E. Roundy, The relationship between the Madden–Julian oscillation and US violent tornado outbreaks in the spring. Mon. Wea. Rev. 141, 2087–2095 (2013).

[15] C. Liu, B. Tian, K.-F. Li, G. L. Manney, N. J. Livesey, Y. L. Yung, D. E. Waliser, Northern Hemisphere mid-winter vortex-displacement and vortex-split stratospheric sudden warmings: Influence of the Madden-Julian oscillation and quasi-biennial oscillation. J. Geophys. Res. Atmos. 119, 12599–12620 (2014).

[16] C. I. Garfinkel, S. B. Feldstein, D. W. Waugh, C. Yoo, S. Lee, Observed connection between stratospheric sudden warmings and the Madden-Julian oscillation. Geophys. Res. Lett. 39, L18807 (2012).

[17] F. Vitart, A. W. Robertson, The sub-seasonal to seasonal prediction project (S2S) and the prediction of extreme events. npj Clim. Atmos. Sci. 1, 3 (2018).

[18] Á. F. Adames, J. M. Wallace, Three-dimensional structure and evolution of the moisture field in the MJO. J. Atmos. Sci. 72, 3733–3754 (2015).

[19] B. O. Wolding, E. D. Maloney, Objective diagnostics and the Madden-Julian oscillation. Part II: Application to moist static energy and moisture budgets. J. Clim. 28, 7786–7808 (2015).

[20] D. Kim, H. Kim, M.-I. Lee, Why does the MJO detour the Maritime Continent during austral summer? Geophys. Res. Lett. 44, 2579–2587 (2017).

[21] D. Kang, D. Kim, M.-S. Ahn, S.-I. An, The role of the background meridional moisture gradient on the propagation of the MJO over the Maritime Continent. J. Clim. 34, 6565–6581 (2021).

[22] B. Wang, Dynamics of tropical low-frequency waves: An analysis of the moist kelvin wave. J. Atmos. Sci. 45, 2051–2065 (1988).

[23] E. D. Maloney, D. L. Hartmann, Frictional moisture convergence in a composite life cycle of the Madden–Julian oscillation. J. Clim. 11, 2387–2403 (1998).

[24] A. Sobel, E. Maloney, Moisture modes and the eastward propagation of the MJO. J. Atmos. Sci. 70, 187–192 (2013).

[25] Á. F. Adames, D. Kim, The MJO as a dispersive, convectively coupled moisture wave: Theory and observations. J. Atmos. Sci. 73, 913–941 (2016).

[26] B. Wang, F. Liu, G. Chen, A trio-interaction theory for Madden–Julian oscillation. Geosci. Lett. 3, 1–16 (2016).

[27] B. Wang, G. Chen, A general theoretical framework for understanding essential dynamics of Madden–Julian oscillation. Clim. Dyn. 49, 2309–2328 (2017).

[28] A. O. Gonzalez, X. Jiang, Winter mean lower tropospheric moisture over the Maritime Continent as a climate model diagnostic metric for the propagation of the Madden-Julian oscillation. Geophys. Res. Lett. 44, 2588–2596 (2017).

[29] B. Wang, S.-S. Lee, D. E. Waliser, C. Zhang, A. Sobel, E. Maloney, T. Li, X. Jiang, K.-J. Ha, Dynamics-oriented diagnostics for the Madden-Julian oscillation. J. Clim. 31, 3117–3135 (2018).

[30] Á. F. Adames, D. Kim, A. H. Sobel, A. Del Genio, J. Wu, Characterization of moist processes associated with changes in the propagation of the MJO with increasing CO_2_. J. Adv. Model. Earth Syst. 9, 2946–2967 (2017). 29497477 10.1002/2017MS001040 PMC5815406

[31] S. S. Rushley, D. Kim, Á. F. Adames, Changes in the MJO under greenhouse gas–induced warming in CMIP5 models. J. Clim. 32, 803–821 (2019). 31048949 10.1175/JCLI-D-18-0437.1 PMC6491051

[32] C. Zhang, J. Ling, Barrier effect of the Indo-Pacific Maritime Continent on the MJO: Perspectives from tracking MJO precipitation. J. Clim. 30, 3439–3459 (2017).

[33] C. A. DeMott, B. O. Wolding, E. D. Maloney, D. A. Randall, Atmospheric mechanisms for MJO decay over the maritime continent. J. Geophys. Res. Atmos. 123, 5188–5204 (2018).

[34] N. Nakazawa, Tropical super clusters within intraseasonal variations over the western Pacific. J. Meteor. Soc. Jpn. 66, 823–839 (1988).

[35] K. Kikuchi, B. Wang, Spatiotemporal wavelet transform and the multiscale behavior of the Madden-Julian oscillation. J. Clim. 23, 3814–3834 (2010).

[36] B. Stevens, S. Adami, T. Ali, H. Anzt, Z. Aslan, S. Attinger, J. Bäck, J. Baehr, P. Bauer, N. Bernier, B. Bishop, H. Bockelmann, S. Bony, G. Brasseur, D. N. Bresch, S. Breyer, G. Brunet, P. L. Buttigieg, J. Cao, C. Castet, Y. Cheng, A. D. Choudhury, D. Coen, S. Crewell, A. Dabholkar, Q. Dai, F. Doblas-Reyes, D. Durran, A. E. Gaidi, C. Ewen, E. Exarchou, V. Eyring, F. Falkinhoff, D. Farrell, P. M. Forster, A. Frassoni, C. Frauen, O. Fuhrer, S. Gani, E. Gerber, D. Goldfarb, J. Grieger, N. Gruber, W. Hazeleger, R. Herken, C. Hewitt, T. Hoefler, H.-H. Hsu, D. Jacob, A. Jahn, C. Jakob, T. Jung, C. Kadow, I.-S. Kang, S. Kang, K. Kashinath, K. K.-V. Königslöw, D. Klocke, U. Kloenne, M. Klöwer, C. Kodama, S. Kollet, T. Kölling, J. Kontkanen, S. Kopp, M. Koran, M. Kulmala, H. Lappalainen, F. Latifi, B. Lawrence, J. Y. Lee, Q. Lejeun, C. Lessig, C. Li, T. Lippert, J. Luterbacher, P. Manninen, J. Marotzke, S. Matsouoka, C. Merchant, P. Messmer, G. Michel, K. Michielsen, T. Miyakawa, J. Müller, R. Munir, S. Narayanasetti, O. Ndiaye, C. Nobre, A. Oberg, R. Oki, T. Özkan-Haller, T. Palmer, S. Posey, A. Prein, O. Primus, M. Pritchard, J. Pullen, D. Putrasahan, J. Quaas, K. Raghavan, V. Ramaswamy, M. Rapp, F. Rauser, M. Reichstein, A. Revi, S. Saluja, M. Satoh, V. Schemann, S. Schemm, C. S. Poberaj, T. Schulthess, C. Senior, J. Shukla, M. Singh, J. Slingo, A. Sobel, S. Solman, J. Spitzer, P. Stier, T. Stocker, S. Strock, H. Su, P. Taalas, J. Taylor, S. Tegtmeier, G. Teutsch, A. Tompkins, U. Ulbrich, P.-L. Vidale, C.-M. Wu, H. Xu, N. Zaki, L. Zanna, T. Zhou, F. Ziemen, Earth virtualization engines (EVE). Earth Syst. Sci. Data 16, 2113–2122 (2024).

[37] H. Miura, M. Satoh, T. Nasuno, A. T. Noda, K. Oouchi, A Madden-Julian oscillation event realistically simulated by a global cloud-resolving model. Science 318, 1763–1765 (2007). 18079399 10.1126/science.1148443

[38] T. Miyakawa, M. Satoh, H. Miura, H. Tomita, H. Yashiro, A. T. Noda, Y. Yamada, C. Kodama, M. Kimoto, K. Yoneyama, Madden–Julian oscillation prediction skill of a new-generation global model demonstrated using a supercomputer. Nat. Commun. 5, 3769 (2014). 24801254 10.1038/ncomms4769 PMC4024761

[39] B. Stevens, M. Satoh, L. Auger, J. Biercamp, C. S. Bretherton, X. Chen, P. D. Düben, F. Judt, M. Khairoutdinov, D. Klocke, C. Kodama, L. Kornblueh, S.-J. Lin, P. Neumann, W. M. Putman, N. Röber, R. Shibuya, B. Vannière, P. L. Vidale, N. Wedi, L. Zhou, DYAMOND: The DYnamics of the Atmospheric general circulation Modeled On Non-hydrostatic Domains. Prog. Earth Planet. Sci. 6, 61 (2019).

[40] H. Tomita, M. Satoh, A new dynamical framework of nonhydrostatic global model using the icosahedral grid. Fluid Dyn. Res. 34, 357–400 (2004).

[41] M. Satoh, H. Tomita, H. Yashiro, H. Miura, C. Kodama, T. Seiki, A. T. Noda, Y. Yamada, D. Goto, M. Sawada, T. Miyoshi, Y. Niwa, M. Hara, T. Ohno, S.-I. Iga, T. Arakawa, T. Inoue, H. Kubokawa, The Non-hydrostatic Icosahedral Atmospheric Model: Description and development. Prog. Earth Planet. Sci. 1, 18 (2014).

[42] C. Zhang, M. Dong, Seasonality in the Madden-Julian oscillation. J. Clim. 17, 3169–3180 (2004).

[43] S. H. Strogatz, *Nonlinear Dynamics and Chaos* (CRC Press, ed. 2, 2018).

[44] P. E. Roundy, W. M. Frank, Effects of low-frequency wave interactions on intraseasonal oscillations. J. Atmos. Sci. 61, 3025–3041 (2004).

[45] H. Masunaga, T. S. L’Ecuyer, C. D. Kummerow, The Madden–Julian oscillation recorded in early observations from the Tropical Rainfall Measuring Mission (TRMM). J. Atmos. Sci. 63, 2777–2794 (2006).

[46] Y. Wei, H.-L. Ren, W. Duan, G. Sun, Westward-propagating disturbances shape diverse MJO propagation. Geophys. Res. Lett. 50, e2023GL104778 (2023).

[47] G. A. Meehl, G. N. Kiladis, K. M. Weickmann, M. Wheeler, D. S. Gutzler, G. P. Compo, Modulation of equatorial subseasonal convective episodes by tropical-extratropical interaction in the Indian and Pacific Ocean regions. J. Geophys. Res. Atmos. 101, 15033–15049 (1996).

[48] R. W. Moore, O. Martius, T. Spengler, The modulation of the subtropical and extratropical atmosphere in the Pacific basin in response to the Madden–Julian oscillation. Mon. Wea. Rev. 138, 2761–2779 (2010).

[49] H. H. Hendon, M. L. Salby, The life cycle of the Madden–Julian oscillation. J. Atmos. Sci. 51, 2225–2237 (1994).

[50] G. Chen, Deciphering chaos in the Madden-Julian oscillation. npj Clim. Atmos. Sci. 7, 311 (2024).

[51] Climate Prediction Center, National Oceanic and Atmospheric Administration, Cold & warm episodes by season (2025); www.cpc.ncep.noaa.gov/products/analysis_monitoring/ensostuff/ONI_v5.php.

[52] D. Takasuka, T. Kohyama, T. Suematsu, H. Miura, ENSO and QBO controls the favorableness of the MJO realization cooperatively. J. Geophys. Res. Atmos. 130, e2024JD042116 (2025).

[53] F. Liu, J. Zhou, B. Wang, J. C.-H. Leung, D. Chen, Z. Lin, I.-S. Kang, Q. Chao, Z. Ke, K. Fan, B. Liu, G. Huang, P.-C. Hsu, W. Dong, Opportunities and barriers for skillful subseasonal prediction of East Asian summer precipitation. Bull. Am. Meteorol. Soc. 105, E2216–E2230 (2024).

[54] Z. K. Martin, E. A. Barnes, E. Maloney, Using simple, explainable neural networks to predict the Madden-Julian oscillation. J. Adv. Model. Earth Syst. 14, e2021MS002774 (2022).

[55] N.-Y. Shin, D. Kim, D. Kang, H. Kim, J.-S. Kug, Deep learning reveals moisture as the primary predictability source of MJO. npj Clim. Atmos. Sci. 7, 11 (2024).

[56] T. Suematsu, K. Nakai, T. Yoneda, D. Takasuka, T. Jinno, Y. Saiki, H. Miura, Machine learning prediction of the MJO extends beyond one month. arXiv:2301.01254 [physics.ao-ph] (2022).

[57] H. Bartana, C. I. Garfinkel, O. Shamir, J. Rao, Projected future changes in equatorial wave spectrum in CMIP6. Clim. Dyn. 60, 3277–3289 (2023).

[58] B. W. Kerns, S. S. Chen, A 20-year climatology of Madden-Julian oscillation convection: Large-scale precipitation tracking from TRMM-GPM rainfall. J. Geophys. Res. Atmos. 125, e2019JD032142 (2020).

[59] H. Tomita, M. Satoh, K. Goto, An optimization of the icosahedral grid modified by spring dynamics. J. Comput. Phys. 183, 307–331 (2002).

[60] D. Takasuka, C. Kodama, T. Suematsu, T. Ohno, Y. Yamada, T. Seiki, H. Yashiro, M. Nakano, H. Miura, A. T. Noda, T. Nasuno, T. Miyakawa, R. Masunaga, How can we improve the seamless representation of climatological statistics and weather toward reliable global k-scale climate simulations? J. Adv. Model. Earth Syst. 16, e2023MS003701 (2024).

[61] S. Kotsuki, K. Terasaki, K. Kanemaru, M. Satoh, T. Kubota, T. Miyoshi, Predictability of record-breaking rainfall in Japan in July 2018: Ensemble forecast experiments with the near-real-time global atmospheric data assimilation system NEXRA. SOLA 15A, 1–7 (2019).

[62] R. W. Reynolds, T. M. Smith, C. Liu, D. B. Chelton, K. S. Casey, M. G. Schlax, Daily high-resolution-blended analyses for sea surface temperature. J. Clim. 20, 5473–5496 (2007).

[63] B. Liebmann, C. Smith, Description of a complete (interpolated) outgoing longwave radiation dataset. Bull. Am. Meterol. Soc. 77, 1275–1277 (1996).

[64] H. Hersbach, B. Bell, P. Berrisford, S. Hirahara, A. Horányi, J. Muñoz-Sabater, J. Nicolas, C. Peubey, R. Radu, D. Schepers, A. Simmons, C. Soci, S. Abdalla, X. Abellan, G. Balsamo, P. Bechtold, G. Biavati, J. Bidlot, M. Bonavita, G. De Chiara, P. Dahlgren, D. Dee, M. Diamantakis, R. Dragani, J. Flemming, R. Forbes, M. Fuentes, A. Geer, L. Haimberger, S. Healy, R. J. Hogan, E. Hólm, M. Janisková, S. Keeley, P. Laloyaux, P. Lopez, C. Lupu, G. Radnoti, P. de Rosnay, I. Rozum, F. Vamborg, S. Villaume, J.-N. Thépaut, The ERA5 global reanalysis. Q. J. Roy. Meteorol. Soc. 146, 1999–2049 (2020).

[65] K. Kikuchi, B. Wang, Y. Kajikawa, Bimodal representation of the tropical intraseasonal oscillation. Clim. Dyn. 38, 1989–2000 (2012).

[66] R. Shibuya, M. Nakano, C. Kodama, T. Nasuno, K. Kikuchi, M. Satoh, H. Miura, T. Miyakawa, Prediction skill of the boreal summer intra-seasonal oscillation in global non-hydrostatic atmospheric model simulations with explicit cloud microphysics. J. Meteor. Soc. Jpn. Ser. 99, 973–992 (2021).

[67] C. E. Duchon, Lanczos filtering in one and two dimensions. J. Appl. Meteorol. 18, 1016–1022 (1979).

[68] B. J. Hoskins, I. Draghici, H. Davies, A new look at the ω-equation. Q. J. Roy. Meteorol. Soc. 104, 31–38 (1978).

[69] K. Takaya, H. Nakamura, A formulation of a phase-independent wave-activity flux for stationary and migratory quasigeostrophic eddies on a zonally varying basic flow. J. Atmos. Sci. 58, 608–627 (2001).

[70] K. Nishii, H. Nakamura, Lower-stratospheric Rossby wave trains in the southern hemisphere: A case-study for late winter of 1997. Q. J. Roy. Meteorol. Soc. 130, 325–345 (2004).

[71] B. J. Hoskins, I. N. James, G. H. White, The shape, propagation and mean-flow interaction of large-scale weather systems. J. Atmos. Sci. 40, 1595–1612 (1983).

[72] D. Takasuka, T. Suematsu, H. Miura, M. Nakano, Propagation of the Madden-Julian oscillation as a deterministic chaotic phenomenon (2025). 10.1126/sciadv.adz1916 PMC12915619 41706853

