# Supplementary material for: Propagation of the Madden-Julian oscillation as a deterministic chaotic phenomenon
Source: Sci Adv. 2026 Feb 18;12(8):eadz1916. doi: 10.1126/sciadv.adz1916 (PMC12915619; doi:10.1126/sciadv.adz1916)
Supplement: Supplementary file 1 — Supplementary Text Figs. S1 to S6 [file sciadv.adz1916_sm.pdf]

Science Advances
AAAS

Supplementary Materials for

Propagation of the Madden-Julian oscillation as a deterministic chaotic phenomenon

Daisuke Takasuka *et al.*

Corresponding author: Daisuke Takasuka, takasuka@tohoku.ac.jp

Sci. Adv. **12**, eadz1916 (2026)
DOI: 10.1126/sciadv.adz1916

This PDF file includes:

Supplementary Text
Figs. S1 to S6

Supplementary Text

Moistening processes supporting the two regimes of the Dec-MJO propagation

Figures 1F, 1G, and 2A clarify the difference in the moisture accumulation over the WP, which separates the MJO propagation features between the Dec-1 and Dec-2 regimes. This difference is quantified by the MSE budget analysis. First, we compare the anomalous MSE budget terms averaged over the northern WP during December 11–15, when the moisture-laden westward-propagating wave survives and decays for the Dec-1 and Dec-2 regime, respectively (fig. S3A). Moisture tendency is much more positive in the Dec-1 regime, supported by the more horizontal advective moistening. This confirms that the synoptic-scale equatorial Rossby wave transports moisture and thus supports the Dec-1 MJO propagation.

Meanwhile, the moisture accumulation contributing to the MJO propagation for the Dec-2 regime (during December 20–24) is driven by the enhanced surface heat fluxes (fig. S3B), as described in the main text. In fact, after December 17 (Fig. 2A), the large-scale convective envelopes begin to be organized over the northern WP, where the surface heat fluxes are prominent significantly (fig. S3C). The enhanced surface heat fluxes result from the superimposition of anomalous easterlies in the Northern Hemisphere and their southeastward redirection toward the Southern Hemisphere onto the background mean flows (fig. S3D).

Processes differentiating the development of the equatorial Rossby wave

We quantify how different the equatorial Rossby wave activities affecting the moistening processes over the WP is between the Dec-1 and Dec-2 regimes. As a reference, Figure S4A shows the domain-mean (0°–15°N, 160°–180°E) vertical profile of the ERA5-derived EKE budget terms on December 7, right before the westward-propagating off-equatorial low-level cyclonic circulations intrude into the target domain (Fig. 1B and fig. S2C). Note that we omit *AeKe* here because of few contributions from it. The positive EKE tendency in the lower troposphere is attributed to *GKe*, suggesting a result of the energy redistribution. Of the two energy sources (i.e., *KmKe* and *PeKe*), the contributions from *PeKe* are more positive in the column than *KmKe*, and they are largest in the upper troposphere. These situations mean that EKE generated by baroclinic conversion is dispersed downward; that is, wave-convection coupling is a dominant process that supports the development of the equatorial Rossby wave. This view is successfully reproduced in the Dec-1 regime (fig. S4B).

Based on the above insight, we compare the domain-mean vertical structure of *PeKe* and *GKe* between the Dec-1 and Dec-2 regimes on December 7 (fig. S4C). Both the upper-level *PeKe* and lower-level *GKe* are more predominant in the Dec-1 regime, as expected. This confirms that the wave-convection coupling is weaker in the Dec-2 regime, consistent with the fact that the Dec-2 regime does not benefit from the off-equatorial dynamical forcing by the extratropical trough intrusion (Fig. 4B).

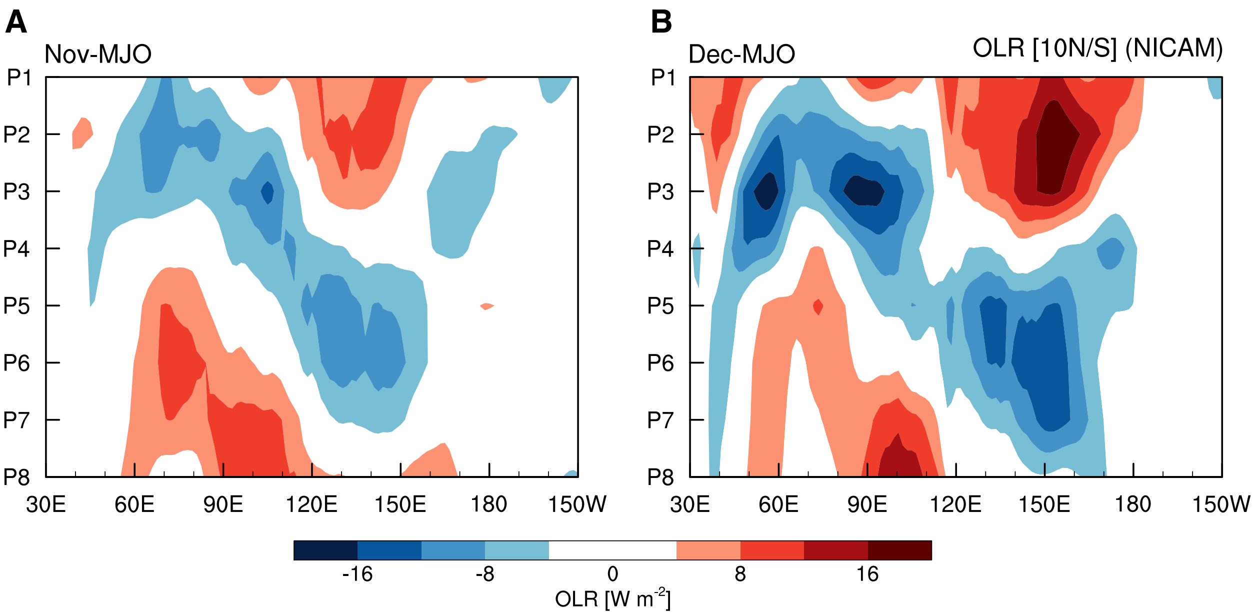

Fig. S1.

Phase composites of the eastward propagation of the simulated two MJO events. Longitudinal distributions of equatorial (10°S–10°N) OLR anomalies composited for each of the eight MJO phases on which the amplitudes A is 0.4 or more from all the ensemble members of the Nov-MJO (**A**) and Dec-MJO simulations (**B**). The x-axis and y-axis denote the longitudes and the phase numbers, respectively.

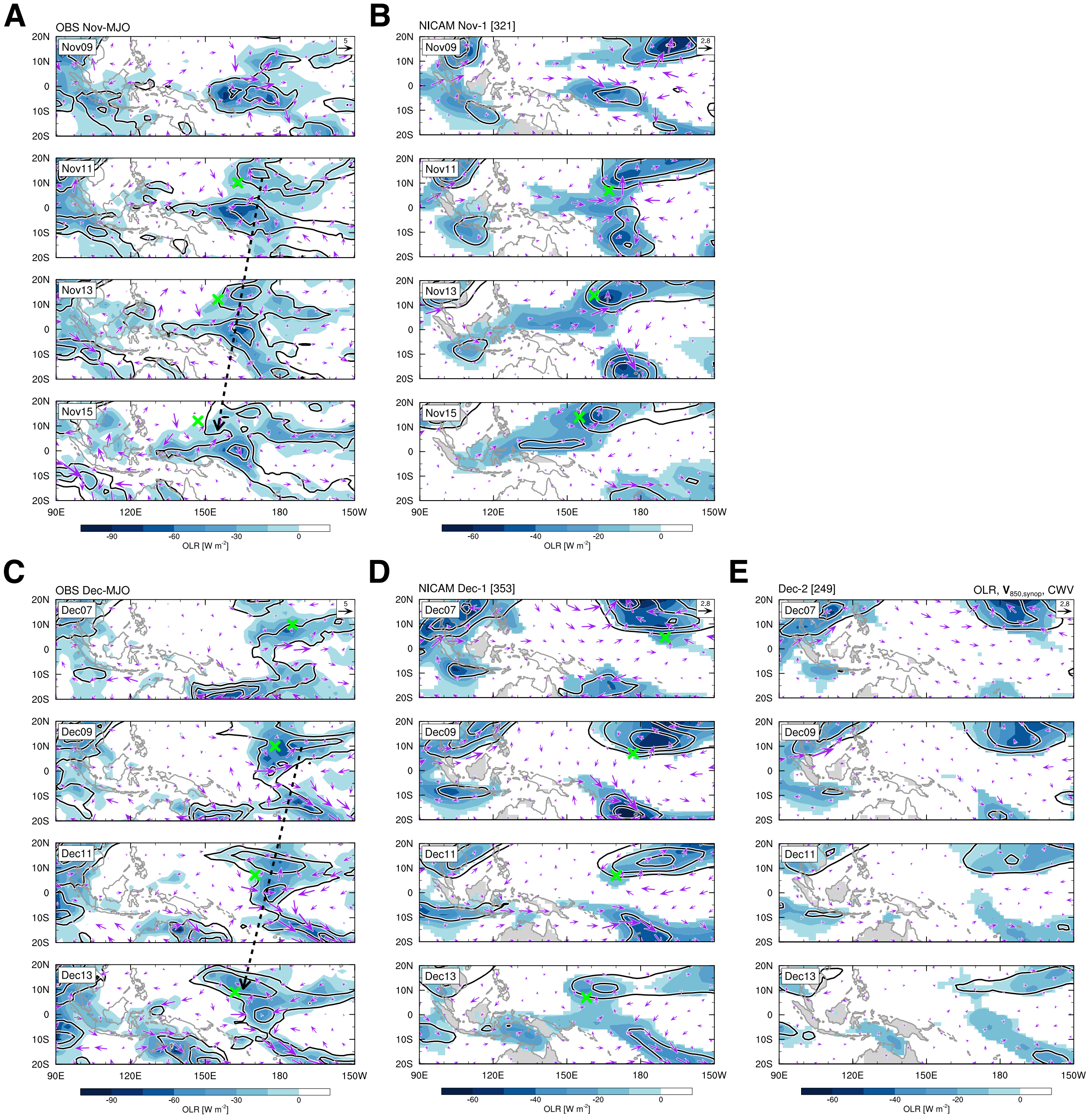

Fig. S2.

Horizontal structure of the westward-propagating synoptic-scale wave affecting the MJO propagation. Spatio-temporal evolution of OLR (shading), column water vapor (contours), and 850-hPa horizontal wind anomalies (vectors). Observations for Nov-MJO (**A**) and Dec-MJO (**C**), and simulations composited for the Nov-1 (**B**), Dec-1 (**D**), and Dec-2 regime (**E**). Contour interval is 6 kg m^{-2}, with zero or less values omitted. Shading and vectors in (**B**, **D**, and **E**) indicate statistical significance at the 99% level. Green cross marks denote the center of the westward-propagating cyclonic circulations, and broken arrows in (**A** and **C**) represent the westward moisture transport.

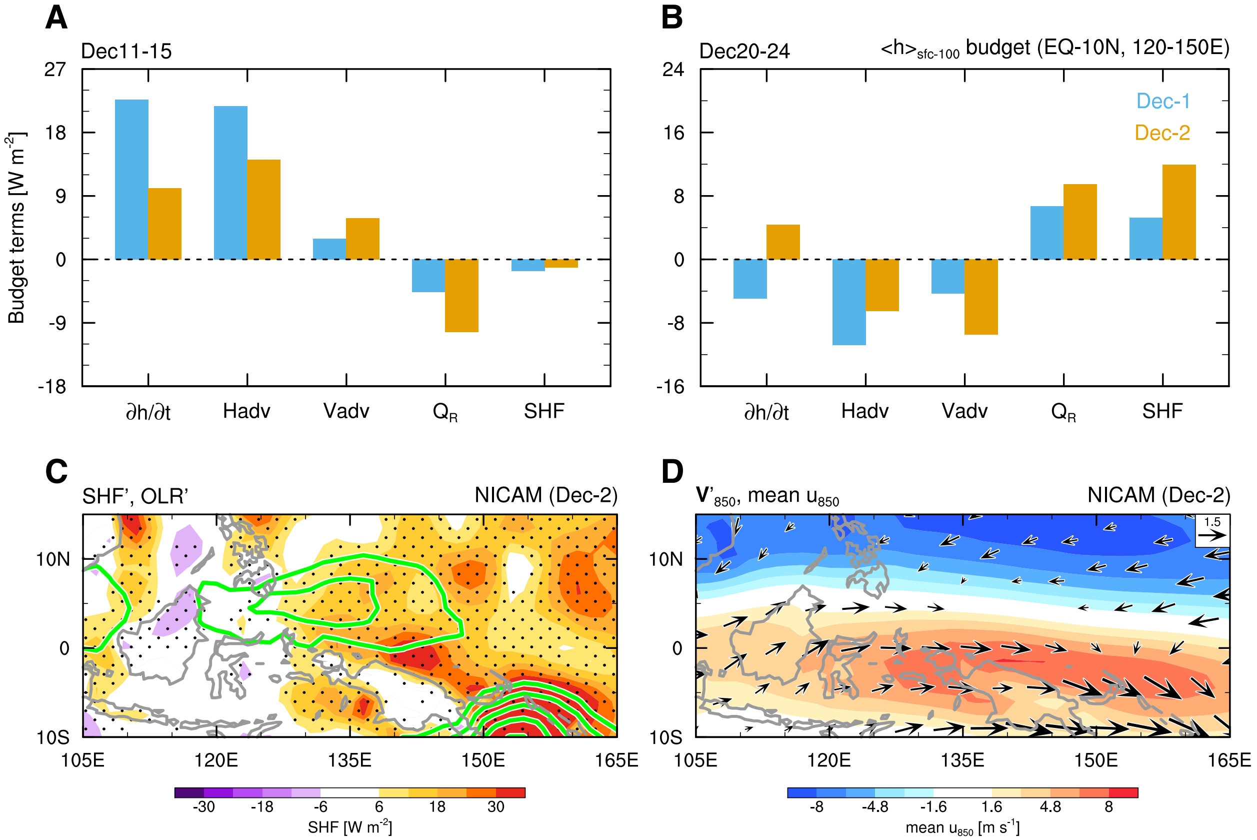

Fig. S3.

Comparisons of the moistening processes over the northern WP between the Dec-1 and Dec-2 regimes. (**A** and **B**) Anomalous column-integrated MSE budget terms averaged over 0°–10°N, 120°–150°E during December 11–15 (**A**) and December 20–24 (**B**), composited for the Dec-1 (blue) and Dec-2 regime (orange). (**C**) Surface heat flux (shading) and OLR (contours) anomalies during December 20–24, composited for the Dec-2 regime. Contours indicate -15 W m^{-2} or less values with their interval of 5 W m^{-2}. Stippling indicates statistical significance at the 99% level. (**D**) As in (C), but for 850-hPa zonal winds averaged over the simulation period (shading) and 850-hPa horizontal wind anomalies during December 20–24. Vectors indicate statistical significance at the 99% level.

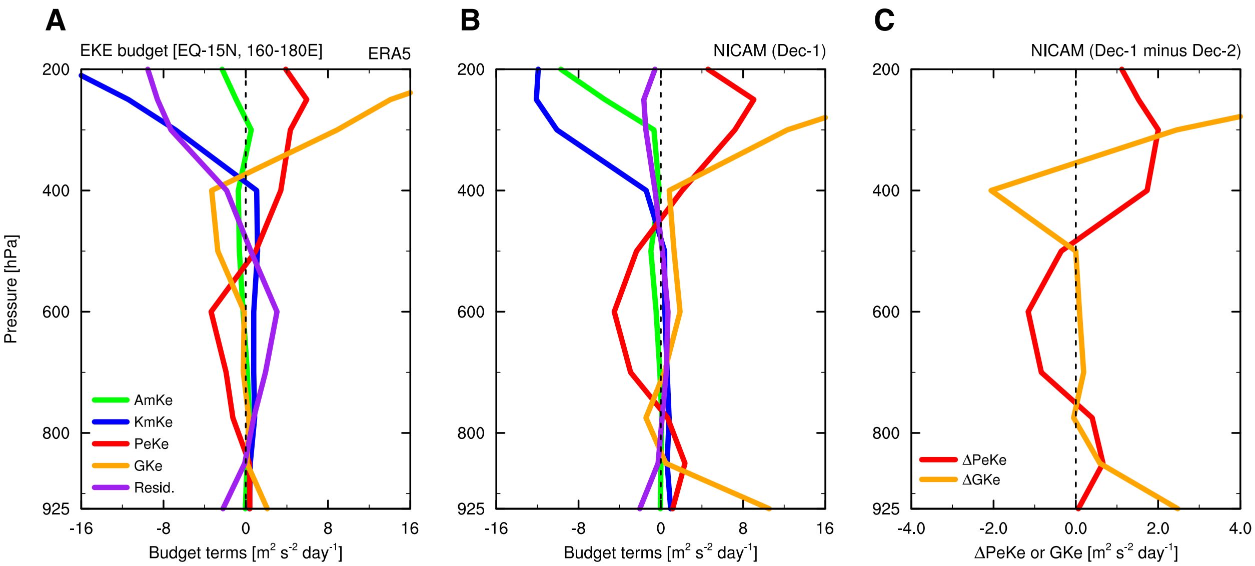

Fig. S4.

EKE budget analysis for the westward-propagating synoptic-scale wave in the observations, Dec-1 and Dec-2 regimes. (**A**) Vertical profile of the five EKE budget terms (*AmKe*, *KmKe*, *PeKe*, *Gke*, and *Residual*) averaged over 0°–15°N, 160°–180°E on December 7 from ERA5. (**B**) Same as (A), except for the Dec-1 regime. (**C**) As in (A), but for the difference of *PeKe* and *GKe* between the Dec-1 and Dec-2 regime.

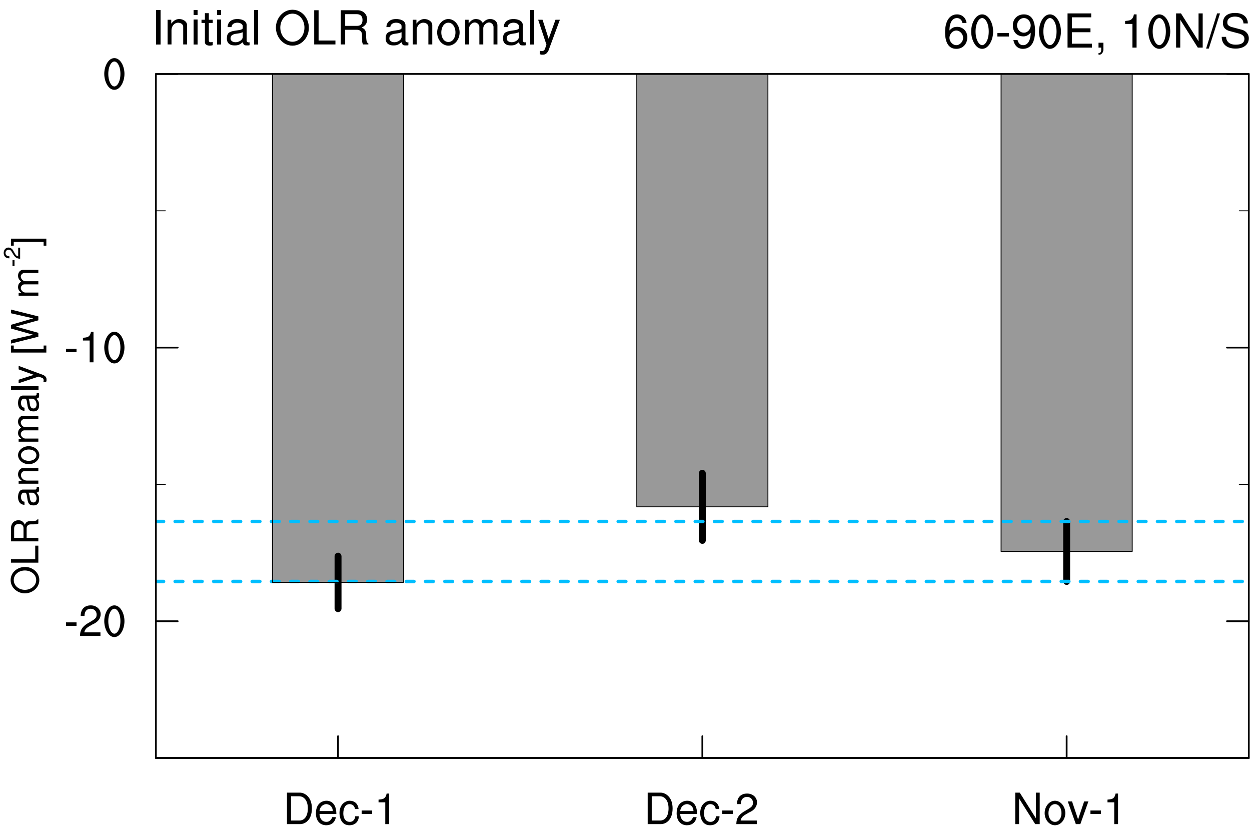

Fig. S5.

Sensitivity of the regime selection to initial amplitudes of MJO convection. Comparisons of composite OLR anomalies averaged over the Indian Ocean (60°–90°E, 10°N/S) during the initial phase of MJO convection between the Dec-1, Dec-2 and Nov-1 regimes. The initial phase is defined as the 5-day period of December 5–9 and November 3–7 for Dec-MJO and Nov-MJO, respectively. Black lines attached with the box plots indicate the 99% confidence interval, and blue-dotted lines represent that interval for the Nov-1 regime.

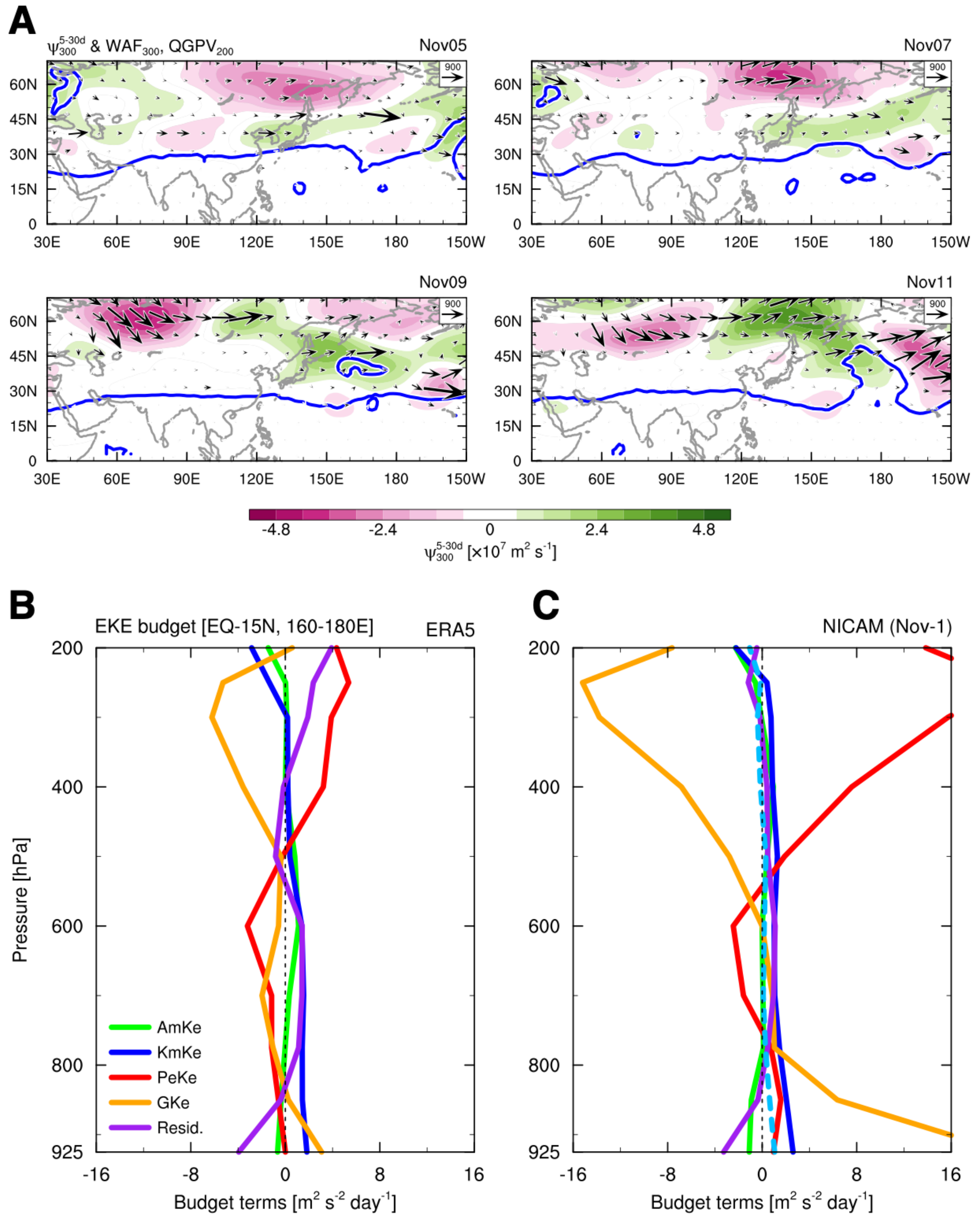

Fig. S6.

Tropical-extratropical dynamic evolution for observed Nov-MJO and/or the Nov-1 regime. (**A**) Observed 300-hPa Takaya-Nakamura wave activity flux (*45*) (vectors), 200-hPa QGPV (contours), and 5–30-day bandpass-filtered 300-hPa stream function (shading) every 2 days from November 5 to 11. Contour represents 5.0×10^{-5} s^{-1}. (**B** and **C**) Vertical profiles of the five EKE budget terms (*AmKe*, *KmKe*, *PeKe*, *Gke*, and *Residual*) averaged over 0°–15°N, 160°–180°E on November 7 from ERA5 (**B**) and for the Nov-1 regime in the simulations (**C**). As exception, a light-blue broken line in (C) indicates *KmKe* for the composite of non-propagating members in the simulations.
